# Supplementary material for: Toward Participatory Precision Health With Co-Designed Recommendations: Systematic Review of Just-in-Time Adaptive Interventions in Adolescents and Young Adults
Source: J Med Internet Res. 2026 May 21;28:e84422. doi: 10.2196/84422 (PMC13193708; doi:10.2196/84422)
Supplement: Multimedia Appendix 1 [file jmir-v28-e84422-s001.pdf]

Data Extraction for Just-In-Time Adaptive Interventions (JITAIs) Addressing Adolescent and Young Adult (AYA) Substance Use

Contents:

Table 1: Study design, theory, and evidence from randomized controlled trials

Table 2: Study design, theory, and evidence from non-controlled pilot studies

Table 3: Study design, theory, and evidence from qualitative studies

Table 4: Tailoring mechanisms of JITAIs evaluated in randomized controlled trials

Table 5: Tailoring mechanisms of JITAIs evaluated in non-controlled pilot studies

Table 6: Tailoring mechanisms of JITAIs evaluated in qualitative studies

NR = not reported

Table 1: Study design, theory, and evidence from randomized controlled trials

Authors, year	Setting	Population (N, condition, age)	Study design	Target outcomes	Theoretical or tailoring framework	Missing data or data completeness	Key outcome pattern
Rodgers et al. 2005	New Zealand	N=1705 smokers wanting to quit, aged 16+; median age 22	Randomized controlled trial, parallel groups, 26-week follow-up; assessments at 6, 12, and 26 weeks	Distal outcome: smoking cessation/current non-smoking; proximal outcomes: NR	NR	Follow-up available for 95.2% at 6 weeks, 89.7% at 12 weeks, and 74.2% at 26 weeks; lower 26-week follow-up in intervention group; missing=smoking in primary analysis	Higher self-reported non-smoking in intervention at 6 and 12 weeks; 26-week differences less clear because of incomplete follow-up
Weitzel et al. 2007	Private university in the southeastern United States	N=40 college students drinking more than once a week; mean age 19.2	Randomized trial of handheld-only monitoring vs handheld monitoring plus tailored messaging; 2 weeks; follow-up at 2 weeks	Distal outcomes: alcohol-related behaviors and consequences; proximal outcomes: alcohol consequence expectancies and self-efficacy	NR	All completed baseline and follow-up; daily adherence about 83.3% to 85.0%; one HHM outlier excluded from between-group analyses; no imputation reported	Fewer drinks per drinking day in treatment on handheld study-period reports and lower trouble expectancies at follow-up; most other effects not significant
Suffoletto et al. 2012	3 urban emergency departments in Western	N=45 hazardous-drinking young adults aged 18 to 24; mean age about 21	Pilot randomized controlled trial, 3 arms, 12 weeks, 3-	Distal outcome: hazardous/heavy drinking reduction; proximal outcomes: weekly heavy drinking, drinks per	NIAAA clinician guide for alcohol brief interventions	87% retained at 3 months; 88% weekly text assessments answered; 77% completed all 12 weeks; unanswered weekly	Intervention reduced heavy drinking days and drinks per drinking day at 3 months, with post hoc

Authors, year	Setting	Population (N, condition, age)	Study design	Target outcomes	Theoretical or tailoring framework	Missing data or data completeness	Key outcome pattern
	Pennsylvania, United States		month follow-up	drinking day, goal setting		data considered lost; no imputation reported	differences vs assessment group only
Witkiewitz et al. 2014	Large public university in the United States	N=94 non-treatment-seeking college students with at least one heavy drinking episode in past 2 weeks and concurrent smoking and drinking at least weekly; mean age 20.5	Randomized controlled trial, 3 arms, 14-day EMA period, 1-month follow-up	Distal outcomes: heavy drinking and smoking reduction; proximal outcomes: real-time drinking, smoking, and concurrent drinking-smoking episodes during EMA	BASICS-derived feedback with cognitive-behavioral elements, relapse prevention, and mindfulness-based relapse prevention	89.4% completed follow-up; 83.5% random EMA completion; 66.7% completed event-contingent EMAs; attrition linked to higher baseline smoking; baseline smoking covaried and sensitivity analysis used baseline smoking imputation for missing follow-up	No significant effects on drinking, heavy drinking, or concurrent use at follow-up; mobile intervention and monitoring reduced cigarettes per smoking day vs minimal assessment; more modules predicted less drinking during EMA and lower smoking at follow-up
Suffoletto et al. 2015	4 emergency departments in Pittsburgh, Pennsylvania, United States	N=765 hazardous-drinking young adults aged 18 to 25; mean age 22.0	Randomized controlled trial, 3 arms, 12-week SMS intervention, follow-up at 3, 6, and 9 months	Distal outcomes: binge drinking reduction and alcohol-related injury reduction; proximal outcomes: weekend drinking intentions, goal commitment, and weekend drinking quantity	Theory of Planned Behavior	Retention 78.2% at 3 months, 63.5% at 6 months, 54.9% at 9 months; Sunday SMS response declined over 12 weeks; about 33% completed all text queries; multiple imputation used	Interactive SMS reduced binge drinking days, binge drinking prevalence, drinks per drinking day, and alcohol-related injury versus control; assessment-only showed no significant benefit

Authors, year	Setting	Population (N, condition, age)	Study design	Target outcomes	Theoretical or tailoring framework	Missing data or data completeness	Key outcome pattern
Shrier et al. 2018	Five primary care clinics affiliated with an urban children's hospital in the United States	N=70 youth using marijuana at least 3 times/week; age 15 to 24; mean age 20.7	Pilot parallel-group randomized trial, 3 arms, 3-month follow-up	Distal outcomes: marijuana use/problems reduction; proximal outcomes: momentary desire and marijuana use in targeted contexts	Motivational enhancement therapy / motivational interviewing	66% completed assigned treatment; 63% completed at least one 3-month assessment overall; EMA response declined across phases; no imputation reported	Across arms, marijuana use, desire, and problems decreased; MOMENT showed greater decline in momentary desire than MET-only, and use after targeted contexts/behaviors declined more in MOMENT and No-messages than MET-only
Wright et al. 2018	Melbourne, Australia; recruited from an observational cohort of young adults	N=269 young adults aged 18 to 29 with recent risky drinking	Three-arm randomized controlled trial: EMI vs EMA-only vs no-contact control; 12-week intervention period with follow-up at 12 weeks	Distal outcomes: peak risky single-occasion drinking and alcohol-related harms; proximal outcomes: event-level drinking, spending, intentions, and adverse-event avoidance	Motivational interviewing and brief intervention theory, informed by FRAMES	87/269 completed follow-up; low uptake/registration; among registrants many completed all 6 events; ITT mixed models with maximum likelihood used for incomplete data; technical issues reported	No significant group differences on peak risky drinking, other alcohol measures, or harms; acceptability was generally high despite technical problems
Suffoletto et al. 2019	Urban emergency department in	N=149 non-treatment-seeking young	Pilot randomized clinical trial; 5	Distal outcomes: binge drinking and drinks per drinking	Behavior change techniques framework	127/149 randomized after run-in; 79% EMA completion overall	EMA outcomes favored COMBO, with significant reductions in binge

Authors, year	Setting	Population (N, condition, age)	Study design	Target outcomes	Theoretical or tailoring framework	Missing data or data completeness	Key outcome pattern
	Pittsburgh, United States	adults with hazardous drinking and at least one binge episode in prior month; 127 randomized after run-in; mean age 21.7	SMS arms; 2-week run-in, 12-week intervention, 14- and 28-week follow-up	day; proximal outcomes: drinking plans, desire to get drunk, goal striving, and post-drinking appraisal		declining from about 93% to 65%; web follow-up 72.4% at 14 weeks and 67.7% at 28 weeks; multiple imputation used in sensitivity analyses	drinking and max drinks per drinking day over time; TLFB outcomes showed limited or mixed differences
O'Donnell et al. 2019	Australia; recruited via social media and a large metropolitan university campus	N=45 young adults motivated to reduce alcohol use, drinking at least weekly, aged 18 to 35	Single-blind randomized controlled pilot trial; intervention app vs self-monitoring control app; 28 days	Distal outcomes: risky single occasion drinking and drinking-related harms; proximal outcomes: protective behavioral strategy use	Protective Behavioral Strategies framework	Follow-up completed by 22/25 intervention and 16/20 control participants; adherence 79% of days in both groups; missing follow-up handled with full-information maximum likelihood after MCAR test	No significant drinking or harm differences; intervention increased protective strategy use and had high usability/acceptability
Santa Maria et al. 2021	Houston, Texas region; shelter and drop-in center serving youth experiencing homelessness	N=97 analyzed young adults experiencing homelessness, age 18 to 25; mean age 21.2	Pilot randomized attention control trial; 6-week smartphone EMA/JITAI	Distal outcomes: HIV risk reduction behaviors; proximal outcomes: daily sexual activity, drug use, alcohol use, urges, and stress; also	Information-Motivation-Behavioral Skills model	Median app engagement 34.5 days; median 19 days of useful EMA data; 46.3% of all possible response days completed; phone loss common; sensitivity	Intervention reduced odds of drug use and urge for sex relative to control; both groups improved over time; no clear intervention effect on alcohol use, sexual

Authors, year	Setting	Population (N, condition, age)	Study design	Target outcomes	Theoretical or tailoring framework	Missing data or data completeness	Key outcome pattern
				PrEP use and HIV testing		analysis addressed missingness	activity, PrEP use, or HIV testing
Lee et al. 2025	Greater Seattle metropolitan area, United States; students at 2- and 4-year colleges during COVID-19	N=408 college students aged 18 to 25 with recent high-risk drinking and consequences; mean age 20.84	Randomized controlled trial of 21-day browser-based mobile JITAI vs assessment-only control; follow-ups at 1, 6, and 12 months	Distal outcomes: drinks/week, HED frequency, peak eBAC, negative consequences, hazardous/harmful drinking; proximal outcomes: alcohol expectancies	Alcohol expectancy theory and social learning/cognitive-behavioral principles within a JITAI	95.6% at 1 month, 89.2% at 6 months, 88.0% at 12 months; no baseline predictors of attrition; maximum likelihood for multilevel models; listwise deletion for AUDIT	Both groups improved; only significant between-group effect was a greater 1-month reduction in HED in intervention; no other significant effects

Table 2: Study design, theory, and evidence from non-controlled pilot studies

Authors, year	Setting	Population (N, condition, age)	Study design	Target outcomes	Theoretical or tailoring framework	Missing data or data completeness	Key outcome pattern
Riley et al. 2008	Large public university in the Washington, DC area	N=31 college smokers aged 18 to 24 who wanted to quit within 30 days	Single-arm pre-post trial; 6-week follow-up	Distal outcome: smoking cessation; proximal outcomes: cigarettes/day and nicotine dependence	Self-regulation and transtheoretical model	87.1% retained at 6 weeks; 4/31 lost; missing counted as smoking	45% reported 7-day abstinence, 42% biochemically verified; continuing smokers reduced cigarettes/day and dependence
Shrier et al. 2014a (same intervention as Shrier et al. 2014b)	Two adolescent clinics affiliated with a pediatric hospital in a Northeast US city	N=27 youth using marijuana at least 3 times/week; median age 19, range 15 to 24	Single-arm pilot; 1-week baseline, 2-week intervention, 3-month follow-up	Distal outcomes: marijuana use/problems; proximal outcomes: momentary desire and marijuana use after trigger exposure	Motivational enhancement therapy with self-efficacy and coping focus	22/27 completed baseline mobile assessment, 16/27 intervention, 15/27 3-month ACASI/TLFB, 14/27 follow-up mobile assessment; no imputation reported	Desire in trigger contexts decreased and odds of use after trigger exposure were lower at follow-up (OR 0.54, 95% CI 0.31 to 0.95); daily/individual outcomes improved non-significantly
Leonard et al. 2017	University health center at a large private	N=10 non-treatment-seeking female undergraduates	Single-arm acceptability/feasibility pilot; baseline and follow-up with about 3	Distal outcomes: reduced risky drinking and related harms; proximal	Motivational interviewing and cognitive behavioral	10/11 enrolled completed baseline and follow-up; app	High acceptability and perceived usefulness; improved awareness/coping

**Authors, year**	**Setting**	**Population (N, condition, age)**	**Study design**	**Target outcomes**	**Theoretical or tailoring framework**	**Missing data or data completeness**	**Key outcome pattern**
	university in the United States	with risky drinking; mean age 20.7, range 19 to 22	to 4 weeks of intervention use	outcomes: awareness of emotions/triggers, coping, and protective drinking strategies	therapy; UTAUT guided technology acceptability assessment	used on 78/292 available days (26.7%); wide variation and technical issues noted	and drinking self-monitoring; not powered for efficacy
Braciszewski et al. 2018	Large New England post-foster care transition services agency, United States	Focus groups N=24; open trial N=17 enrolled, N=16 analyzed; age 18 to 19	Mixed methods development study with focus groups and 6-month single-arm open trial; follow-ups at 3 and 6 months	Distal outcomes: substance use reduction, especially percent days abstinent; proximal outcomes: readiness to change and weekly substance use	Motivational interviewing and FRAMES for SBI; transtheoretical model for text tailoring	59% retained at 3 and 6 months; weekly poll response 82%; ITT HLM used all available data with LOCF sensitivity analysis	Percent days abstinent increased at 1 month but not significantly; no linear change over 6 months; feasibility and acceptability were high
Suffoletto et al. 2018	Urban emergency department, United States	N=50 young adults aged 18 to 25 with hazardous drinking and at least 1 binge episode in prior month; N=38 enrolled after run-in	Nonrandomized clinical trial with 2-week run-in, voluntary 4-week intervention blocks, and 3-month follow-up	Distal outcomes: reduced weekend alcohol use and alcohol-related consequences; proximal outcomes: weekend plans, goal commitment, self-efficacy, and	Harm reduction, self-regulation, and behavioral shaping	38/50 enrolled after run-in; 37/50 completed 3-month follow-up; EMA response averaged 82.3%, 75.3%, and 72.8% across first 3 blocks; no	High engagement, goal commitment, and goal success; weekend drinking and consequences decreased, but reductions did not differ by length of exposure

**Authors, year**	**Setting**	**Population (N, condition, age)**	**Study design**	**Target outcomes**	**Theoretical or tailoring framework**	**Missing data or data completeness**	**Key outcome pattern**
				maximum weekend drinks		imputation reported	
Stevenson et al. 2020 and Blevins et al. 2021	Large private psychiatric partial hospitalization program in New England, United States	N=20 emerging adults aged 18 to 26 with anxiety and/or depression symptoms who reported drinking to cope	Single-arm open pilot; 6-week EMA/EMI plus in-person feedback/orientation	Distal outcomes: alcohol use, alcohol-related problems, and drinking-to-cope motives; proximal outcomes: negative mood, coping strategy use, and same-day drinking	Personalized feedback on alcohol use/coping motives with coping-skills relapse prevention	75% completed 6-week follow-up; EMA completion declined over time; overall 52.9% of prompts completed; no imputation reported	Alcohol use, binge drinking, alcohol problems, and coping motives decreased over time; coping increased, and greater pre-drinking coping predicted lower alcohol use when negative mood was high
Kazemi et al. 2020 (Study 2)	Large public university in the southeastern United States	Voluntary undergraduate psychology students reporting alcohol use in the past month; AO control n=157, app n=81; mean age about 19.9	Nonrandomized controlled trial; assessment-only control vs BMI+SP app; 2-week app; 6-week follow-up	Distal outcomes: hazardous drinking, peak BAC, and alcohol consequences; proximal outcomes: protective behavioral strategies and readiness to change	Transtheoretical model, motivational interviewing, and ecological momentary intervention principles	6-week follow-up 64.3% in control and 85.2% in app; app users received about 74 messages and opened about 66 on average; no imputation, per-protocol analyses	App group showed significant reductions in AUDIT scores and alcohol consequences; control showed no significant changes

Authors, year	Setting	Population (N, condition, age)	Study design	Target outcomes	Theoretical or tailoring framework	Missing data or data completeness	Key outcome pattern
Coughlin et al. 2021	United States; emergency-department recruited community sample	N=51 emerging adults aged 17 to 24 with past-month alcohol use; mean age 20.47	Secondary analysis of 30-day intensive longitudinal data to develop prediction models for next-day drinking	Distal outcome: reduced risky drinking and consequences; proximal outcome: identification of next-day drinking risk	NR, based on prediction modeling	Mean 20.76/30 daily surveys completed; 30.8% of daily process measures missing; incomplete participant-days removed from GEE analyses	Preferred model predicted next-day drinking with AUC 0.76 using day of week, stress, hopefulness, and sex; alternative model AUC 0.71

Table 3: Study design, theory, and evidence from qualitative studies

Authors, year	Setting	Population (N, condition, age)	Study design	Target outcomes	Theoretical or tailoring framework	Missing data or data completeness	Key study observations
Shrier et al. 2014b (same intervention as Shrier et al. 2014a)	Boston, United States; two adolescent/young adult clinics affiliated with an urban pediatric hospital	N=8 youth using marijuana frequently, age 15 to 24; and N=6 providers	Qualitative formative interviews on the proposed MOMENT intervention	See Shrier et al. 2014a	Motivational enhancement therapy and harm reduction	NR	Generally acceptable and promising; support for immediacy and personalization, with concerns about burden, repetition, carrying the device, signal annoyance, and possible tracking/surveillance
Wright et al. 2016	Melbourne, Australia	N=40 young adults aged 18 to 25 who drank at least weekly	Mixed-methods participatory development study with single-night pilot testing and follow-up evaluation	Distal outcomes: reduced risky single-occasion drinking and related harms; proximal outcomes: real-time awareness of drinking/spending, goal tracking, and safer decision-making during events	Motivational interviewing and brief intervention principles with harm reduction focus	95% retained; 262/295 surveys completed (88.8%); no imputation reported	High acceptability and low intrusion; tracking and tailored feedback increased awareness and sometimes prompted reduced drinking/spending
Cerrada et al. 2017	Los Angeles, United States	Korean American emerging adult smokers aged 18 to 25;	Formative mixed-methods JITAI development paper using	Distal outcome: long-term smoking abstinence; proximal outcome: momentary	Implementation intentions and cultural tailoring for Korean	NA	Development paper proposing JITAI delivering components, see Table 6

**Authors, year**	**Setting**	**Population (N, condition, age)**	**Study design**	**Target outcomes**	**Theoretical or tailoring framework**	**Missing data or data completeness**	**Key study observations**
		interview subsample n=8 and focus group n=4, informed by a prior EMA sample of n=78 daily smokers	prior EMA findings plus interviews and focus group	lapse avoidance in high-risk smoking situations	American emerging adults		
Kazemi et al. 2018	United States, large university setting	N=26 college students in focus groups who reported risky/heavy episodic drinking and had completed an in-person intervention	Intervention development paper using the IDEAS framework	Distal outcome: reduced heavy episodic drinking and alcohol-related problems; proximal outcomes: self-monitoring, motivation/readiness to change, protective strategy use, and safer drinking decisions	Motivational interviewing and transtheoretical model	NR	Development paper describing app modules and tailoring, see Table 6
Acorda et al. 2021	Large urban Southwest U.S. city; shelters and drop-in centers serving youth experiencing homelessness	N=16 youth experiencing homelessness, age 18 to 25	Qualitative exit interviews nested within a pilot randomized trial	Distal outcomes: reduced HIV risk behaviors; proximal outcomes: HIV prevention knowledge, motivation/support, and behavioral skills for safer sex, reduced	Information-Motivation-Behavioral Skills model	NA	Youth described the app as acceptable, informative, motivating, and supportive; they valued goal tracking and personalization, but noted redundancy, timing burden, glitches, and

Authors, year	Setting	Population (N, condition, age)	Study design	Target outcomes	Theoretical or tailoring framework	Missing data or data completeness	Key study observations
				substance use, PrEP interest, and HIV/STI testing			lost/stolen phones, and wanted more tailored messages/questions and stronger real-time support
van Keulen et al. 2022	Netherlands, MBO schools	Dutch lower-educated students aged 16 to 24; pretests across 4 iterations included N=88 total	Development paper using intervention mapping and user-centered design; randomized controlled trial planned	Distal outcome: reduced excessive drinking within Dutch low-risk limits; proximal outcomes: deciding to reduce drinking, setting realistic goals, using effective strategies, monitoring drinking, and evaluating/adjusting goals	Self-determination theory, self-regulation, and intervention mapping	Pretests showed positive usability across iterations; no trial outcome/completion data yet	Development/protocol paper describing a dynamically tailored alcohol-reduction app refined across 4 pretest iterations, see Table 6

Table 4: Tailoring mechanisms of JITAIs evaluated in randomized controlled trials

Authors, year	Intervention options	Tailoring variables and decision rules	Decision points	Human support	Passive sensing	Ethics or youth involvement
Rodgers et al. 2005	Personalised SMS advice/support/distraction; Quit buddy; on-demand craving texts; TXT polls/quizzes; maintenance messages	Preferences, smoking history, barriers to cessation, nickname, and quit date used in a keyword-matching algorithm to tailor message content/programme	Five/day in week before quit date and following 4 weeks, then three/week to 26 weeks; on-demand when user requested craving help	Self-guided only	No	Co-developed with young adults; no specific privacy/ethics tailoring features reported
Weitzel et al. 2007	Daily tailored handheld messages about avoiding alcohol-related consequences	Baseline expectancies and self-efficacy plus daily drinking/consequence reports used for tailoring; messages selected by drinking situation and risk profile	After daily survey completion, if submitted by 5 PM	Yes, staff sent the tailored message once selected by the tailoring matrix	No	NR
Suffoletto et al. 2012	Weekly automated drinking assessments plus tailored feedback, goal prompts, and strategy/reflection messages	Weekly self-reported drinking, sex-specific heavy drinking thresholds, and goal-setting response determined feedback pathway; heavy drinking triggered goal prompt, then strategy if yes and reflection if no	Weekly at noon on participant's typical hangover day; repeat prompt after 6 hours if no reply	No	No	Privacy concerns about texting sensitive information noted; no youth co-design reported

Authors, year	Intervention options	Tailoring variables and decision rules	Decision points	Human support	Passive sensing	Ethics or youth involvement
Witkiewitz et al. 2014	Up to 31 mobile modules including normative feedback, health information, protective strategies, alternative activities, urge-surfing, and decisional balance	Baseline responses personalized module content; real-time urge to smoke triggered urge-surfing content	After each completed random or event-contingent EMA	No	No	NR
Suffoletto et al. 2015	Thursday planning/goal texts plus Sunday drinking assessment and tailored feedback	Weekend drinking plans, willingness to set a below-binge goal, and weekend drinking quantity drove feedback content	Thursday and Sunday each week for 12 weeks	No	No	Young adult input used in refinement; no specific privacy/codesign ethics beyond that reported
Shrier et al. 2018	Two MET sessions plus EMA with responsive motivational messages	Top-3 triggers, momentary desire, recent use, and effort to avoid use triggered messages	After random EMA reports during the 2-week intervention when a targeted trigger/state/behavior was reported	Yes, counselor MET sessions plus RA check-in for low response	No	Waiver of parental permission, Certificate of Confidentiality, confidentiality procedures, and prior youth/provider input into message development
Wright et al. 2018	Event-based EMA with immediate tailored SMS feedback during drinking events	Tailored using gender, time, location, drunkenness, motivation, eating status, cumulative consumption/spending, and intended harms to avoid;	6 pm presurvey, hourly 7 pm to 2 am, and 11 am next day during registered event nights	No	No	Extensive participatory co-design with young people

Authors, year	Intervention options	Tailoring variables and decision rules	Decision points	Human support	Passive sensing	Ethics or youth involvement
		messages mapped to hourly EMA responses/context				
Suffoletto et al. 2019	TRACK, PLAN, USE, GOAL, and COMBO SMS conditions	Baseline drinking pattern determined prompt timing; plans and desire to get drunk guided PLAN feedback; reported consumption guided USE feedback; GOAL limits tailored to past 2-week drinking; COMBO combined all components	Before and after typical drinking occasions on 1 to 3 days per week based on baseline drinking pattern	No	No	Certificate of Confidentiality reported; no youth co-design reported
O'Donnell et al. 2019	App-based self-monitoring with tailored protective strategies, three delivered per drinking event	User goals, affect, and social context guided strategy selection; strategies delivered when user reported drinking or intending to drink	Twice-daily prompts at 11:00 a.m. and 8:00 p.m. for 28 days, with immediate strategy prompt after drinking/intention report	No	No	End-user usability feedback collected; confidentiality/accessibility emphasized
Santa Maria et al. 2021	EMA-driven HIV prevention app with tailored messages and goal-attainment feedback	Baseline HIV prevention goal and EMA-assessed current risk factors such as stress, sexual urge, and substance use were used by an algorithm to select messages across safer sex, substance use, PrEP, and HIV testing domains	After each completed EMA, tapering from 3/day to 2/day to 1/day across 6 weeks	No	No	Extensive youth co-development and beta testing of messages

Authors, year	Intervention options	Tailoring variables and decision rules	Decision points	Human support	Passive sensing	Ethics or youth involvement
Lee et al. 2025	21-day browser-based app with twice-daily surveys; intervention group also received tailored afternoon messages, mostly generic morning psychoeducation, summaries, videos, and tools	Same-day drinking intentions, expected alcohol effects, and context tailored afternoon messages within 7 themes; if no survey completed, non-tailored message shown; morning messages mostly non-tailored	Twice daily in self-selected morning and afternoon windows for 21 days; summaries weekly and at day 21	No	No	NR

Table 5: Tailoring mechanisms of JITAIs evaluated in non-controlled pilot studies

Authors, year	Intervention options	Tailoring variables and decision rules	Decision points	Human support	Passive sensing	Ethics or youth involvement
Riley et al. 2008	Web program plus 1 to 3 tailored SMS/day; quit-day, maintenance, and on-demand S.O.S. coping texts	Quit date, stage of change, and user-identified smoking cues/high-risk times tailored timing and content; slips increased message frequency	Before quit day, on quit day, during maintenance at predicted high-risk times, and on-demand	No	No	NR
Shrier et al. 2014a (same intervention as Shrier et al. 2014b)	Two MET sessions plus 2 weeks of mobile monitoring with responsive messages; extra messages after daily diary use reports	Top-3 triggers, desire to use, and recent use triggered self-efficacy/coping messages; daily diary use also triggered messages	4 to 6 prompts/day plus daily diary; immediate messaging after eligible reports	Yes, two counselor sessions; no ongoing coaching otherwise	No	Waiver of parental consent, Certificate of Confidentiality, and prior youth/provider input into message development
Leonard et al. 2017	Two brief counseling sessions plus app and wearable sensorband with sensor-triggered or self-initiated coping/protective-strategy support	Individual EDA threshold triggered alerts; emotion valence, intensity, context, and drinking intention guided coping and drinking-strategy content	Real-time when EDA crossed threshold, plus self-initiated entries anytime	No	Yes, wearable EDA sensing; accelerometer and temperature supported interpretation	Youth input informed app content
Braciszewski et al. 2018	20-minute computerized brief intervention plus tailored text messages for 6	Drug of choice and readiness to change selected initial content; weekly polls on use and	After baseline SBI, then scheduled texts over 6 months	No	No	Youth input via focus groups and card sorting; privacy/confidentiality discussed

Authors, year	Intervention options	Tailoring variables and decision rules	Decision points	Human support	Passive sensing	Ethics or youth involvement
	months; daily then every other day; weekly polls	readiness updated stage and shifted messages	with weekly poll-based adaptation			
Suffoletto et al. 2018	SMS with adaptive drinking-limit goals, weekend reminders, confidence prompts, and tailored Sunday feedback	Past 2-week maximum weekend drinking set goals; drinking plans triggered goal prompts; confidence ratings tailored support; Sunday maximum drinks determined success/failure feedback	Thursday, Friday, Saturday, and Sunday each week during selected 4-week blocks	No	No	NR
Stevenson et al. 2020 and Blevins et al. 2021	One in-person personalized feedback/orientation session, then 6 weeks of app-based EMA with immediate tailored coping messages	Negative affect plus intent to drink triggered EMI; messages used participant-selected coping strategies generated at baseline	Four random EMA prompts/day across four 3-hour blocks; immediate message after qualifying reports; on-demand EMA available	Yes, one clinician session; no ongoing app counseling	No	Participant-generated coping strategies used for personalization; no youth co-design reported
Kazemi et al. 2020 (Study 2)	BMI+SP app with coach dialogues, personalized feedback, protective strategies, BAC calculator, daily log, education, and resource links	Baseline responses generated personalized feedback and tailored coach messages based on motivation to reduce drinking; users could	During the 2-week app period via daily messages and on-demand tool use	No	No	NR

Authors, year	Intervention options	Tailoring variables and decision rules	Decision points	Human support	Passive sensing	Ethics or youth involvement
		select protective strategies				
Coughlin et al. 2021	SARA app collected daily process measures and weekly 7-day TLFB; no alcohol-reduction intervention content	Prediction rules used day of week, stress, hopefulness, and sex in the preferred model; alternative model used day of week and deviation in hopefulness	Daily evening survey between 6 PM and 12 AM for 30 days, plus weekly Sunday TLFB	No	No	NR

Table 6: Mechanisms of JITAIs developed in qualitative studies

Authors, year	Intervention options	Tailoring variables and decision rules	Decision points	Human support	Passive sensing	Ethics or youth involvement
Shrier et al. 2014b (same intervention as Shrier et al. 2014a)	Proposed intervention with 2 MET sessions, mobile self-monitoring, and responsive messages; extra messages after daily diary use reports	Top 3 triggers, desire to use, recent use, and avoided use triggered messages	4 to 6 random prompts/day plus daily diary for 2 weeks; immediate messaging after eligible reports	Yes, counselor sessions with mobile between-session support	No	Waiver of parental consent, Certificate of Confidentiality, and youth/provider input into intervention development
Wright et al. 2016	Pre-survey, hourly mobile surveys during one drinking event, tailored feedback SMS after each survey, and next-day summary feedback	Gender, goals/plans, drinks consumed, spending, location, priorities/motivations to drink less, and optional self-message guided manually tailored feedback	Pre-survey at nominated start time, hourly during the event until 2 am, and next-day follow-up at 12 pm; feedback after each completed survey	No	No	Extensive co-design with young adults via workshops, testing, and interviews
Cerrada et al. 2017	App modules for MyPlans, MyCalendar, MyCrave, MySmoke, and MyProgress, delivering personalized implementation-intention reminders, craving help, self-monitoring, and progress feedback	User-specified high-risk smoking situations and situation type guided delivery; hourly rule randomized delivery of paired implementation-intention reminders when risk was present, otherwise no reminder	Hourly, assessed 10 minutes before each hour for scheduled high-risk situations; on-demand support via MyCrave	No	No	Participants informed tailoring variables and message content
Kazemi et al. 2018	App prototype with virtual coach dialogues, personalized feedback,	Baseline alcohol history, motivation, triggers/barriers, norms, risk factors, and stage of	Baseline assessment, daily self-monitoring/logging, and ongoing static and	No	No	Focus group input shaped features/content;

Authors, year	Intervention options	Tailoring variables and decision rules	Decision points	Human support	Passive sensing	Ethics or youth involvement
	drink logs, BAC calculator, goals/strategies, educational games, and resources	change tailored feedback and coach language; daily logs and user responses drove dynamic messages	dynamic text prompts during the planned 2-week intervention			privacy concerns discussed
Acorda et al. 2021	App delivered 1 to 4 daily EMA prompts, targeted HIV prevention messages, and goal-progress display; control received generic wellness messages	Youth selected an HIV prevention goal at baseline; EMA reports on feelings, stress, urges, environmental factors, and risk behaviors informed targeted messages and updated progress	1 to 4 prompts/day for up to 6 weeks; message after EMA completion	No	No	YEH co-developed messages and provided refinement input; confidentiality protections for app data described
van Keulen et al. 2022	Standalone native app with 10 sessions over 18 weeks, mini-interventions, role-model videos, daily diary, weekly feedback, and push notifications	Diary alcohol use, mood, importance, confidence, and goal attainment tailored content; lapses, low mood, low motivation/confidence, and noncompletion triggered additional content	Session invitations across 18 weeks, daily diary prompts when goal active, and adaptive push notifications	No	No	Extensive co-design with MBO students and stakeholder advisory involvement
