# Supplementary material for: Toward Participatory Precision Health With Co-Designed Recommendations: Systematic Review of Just-in-Time Adaptive Interventions in Adolescents and Young Adults
Source: J Med Internet Res. 2026 May 21;28:e84422. doi: 10.2196/84422 (PMC13193708; doi:10.2196/84422)
Supplement: Multimedia Appendix 2 [file jmir-v28-e84422-s002.pdf]

Data Extraction for Just-In-Time Adaptive Interventions (JITAIs) Addressing Adolescent and Young Adult (AYA) Mental Health

Contents:

Table 1: Study design, theory, and evidence from randomized controlled trials

Table 2: Study design, theory, and evidence from non-controlled pilot studies

Table 3: Study design, theory, and evidence from qualitative studies

Table 4: Tailoring mechanisms of JITAIs evaluated in randomized controlled trials

Table 5: Tailoring mechanisms of JITAIs evaluated in non-controlled pilot studies

Table 6: Tailoring mechanisms of JITAIs evaluated in qualitative studies

NR = not reported

Table 1: Study design, theory, and evidence from randomized controlled trials

Authors, year	Setting	Population (N, condition, age)	Study design	Target outcomes	Theoretical or tailoring framework	Missing data or data completeness	Key outcome pattern
Kennard et al. 2018	United States, psychiatric inpatient units at 2 academic medical centers	N=66 suicidal adolescents aged 12 to 18; mean age 15.1	Randomized controlled pilot trial; ASAP + treatment as usual vs treatment as usual; follow-up at 4, 12, and 24 weeks postdischarge	Distal outcome: reduced post-discharge suicide attempts; proximal outcomes: emotion regulation, distress tolerance, social support, reasons for living, and safety-plan use	Motivational interviewing, emotion regulation, distress tolerance, and safety planning	60/66 had at least 1 follow-up (90.9%); 6 had no follow-up; blinded independent assessments; ITT analyses used all randomized participants	No significant main effect on attempts or ideation, but attempt rates were lower and time to attempt longer in ASAP; stronger effect among youth with prior attempts
van Aubel et al. 2020	Netherlands; Maastricht region/community recruitment	N=55 randomized, final analyzed N=53; age 16 to 25; subthreshold depressive and/or psychotic symptoms	Single-blind two-arm randomized controlled trial; post, 6-month, and 12-month follow-up	Distal outcomes: reduced subthreshold depression and psychotic-distress symptoms; proximal outcomes: psychological flexibility and daily-life affect	Acceptance and Commitment Therapy in Daily Life (ACT-DL)	100% completed post-measurement among starters; attrition at 6 and 12 months reported; app adherence 48% of beeps; mixed models used REML assuming MAR	Interviewer-rated depression improved more in ACT-DL; self-reported symptoms improved in both groups; daily-life negative affect increased in ACT-DL
Suffoletto et al. 2021	United States, Pittsburgh; recruitment from one primary care	N=52 youth aged 18+ transitioning to college; mean age 18.7; current mental	Pilot randomized controlled trial; 2:1 MoST-MH vs enhanced usual	Distal outcomes: reduced mental health symptom severity and	Positive psychology, cognitive behavioral	94% completed 1-month, 90% 2-month, and 87% 3-month follow-up;	High engagement and usability; completer analyses showed greater

Authors, year	Setting	Population (N, condition, age)	Study design	Target outcomes	Theoretical or tailoring framework	Missing data or data completeness	Key outcome pattern
	clinic and one mental health clinic	health diagnosis or recent mental health care	care; monthly assessments over 3 months	improved functioning during transition to college; proximal outcomes: emotional health awareness, self-management/self-efficacy, stressor recognition, and help-seeking	therapy, and dialectical behavior therapy skills	text check-ins 100% and web check-ins 78%; primary analyses used listwise deletion with multiple-imputation sensitivity analyses	reduction in depressive symptoms with MoST-MH, but no clear differences in self-efficacy or care use, and imputed analyses showed no significant treatment effects
Paetzold et al. 2022 and Reininghaus et al. 2023	Mannheim, Germany	N=92 youth aged 14 to 25 with psychological distress, CHARMS, or first episode of severe mental disorder; mean age 21.67	Assessor-blind exploratory parallel-group randomized controlled trial	Distal outcomes: psychological distress, general psychopathology, and quality of life; proximal outcomes: EMA stress reactivity, momentary resilience, self-compassion, and emotion regulation	Transdiagnostic compassion-focused intervention	98% had outcome data at at least 1 time point; session attendance 96%; mean 14.62 EMI tasks/person/week; EMA compliance high; ITT used all randomized participants	No signal for self-reported distress, but initial signals favored EMIcompass for reduced stress reactivity, lower aberrant salience, higher momentary resilience, improved quality of life, and possibly lower observer-rated symptom

Authors, year	Setting	Population (N, condition, age)	Study design	Target outcomes	Theoretical or tailoring framework	Missing data or data completeness	Key outcome pattern
Schenkel et al. 2022	Switzerland; apprentices in the ready4life program	N=386 apprentices; mean age 17.54 (SD 1.92); 71.5% female	AB/BA crossover trial within ready4life; randomized to planning vs assessment-only order	Distal outcome: reduced occupational stress; proximal outcome: lower same-day perceived stress on stressful apprenticeship days	Just-in-time planning intervention using implementation intentions	Very low field response led protocol change from 6 to 2 measurement points; nonresponders within 6 hours received no later messages; no imputation described	No significant effect of planning on occupational stress; no moderation by vigor or exhaustion
Marciniak et al. 2023	Switzerland; universities in the greater Zurich area	N=95 healthy students with lowered reward sensitivity; mean age 21.5	Two-arm randomized controlled trial; 7-day intervention with baseline and 1-week follow-up	Distal outcomes: depressive symptoms, perceived stress, and anxiety; proximal outcome: reward sensitivity	Mental imagery training targeting reward sensitivity	95/99 randomized completed the study; intervention adherence 88% and control adherence 99%; completer analysis used participants who finished the study	Depressive symptoms and perceived stress improved versus control; anxiety showed a group effect without clear IG pre-post reduction; reward sensitivity did not show clear between-group change
Bell et al. 2023	Australia; online recruitment via social media	N=55 young people aged 16 to 25 with clinical depression,	Open-label parallel-group pilot randomized	Distal outcomes: depression and anxiety; proximal	Transdiagnostic JITAI targeting repetitive	95% completed 6-week assessment overall; 97%	Greater reductions in anxiety and repetitive negative

Authors, year	Setting	Population (N, condition, age)	Study design	Target outcomes	Theoretical or tailoring framework	Missing data or data completeness	Key outcome pattern
		anxiety, and elevated repetitive negative thinking; mean age 20.6	controlled trial; 6-week intervention with assessments at 3 and 6 weeks	outcome: repetitive negative thinking	negative thinking using second- and third-wave CBT techniques	retention in Mello and 92% in control; 82% of weekly phone calls completed; ITT mixed-model analyses used	thinking vs control; depression showed a trend; mediation analyses suggested RNT explained anxiety and depression change; no serious trial-related adverse events
Lucas-Thompson et al. 2023 (same intervention as Lucas-Thompason et al. 2020)	Midwestern United States university	University students with stress/anxiety and smartphone access; attended at least 1 session n=62, analyzed n=50; mean age 21.12	Randomized feasibility trial of a 6-week group mindfulness program with randomized between-session supports; post-test at 6 weeks	Distal outcomes: depression, anxiety, perceived stress, and overall mental health symptoms; proximal outcomes: mindful attention and self-compassion	Mindfulness	80% of session attendees completed post-test (50/62); 979 EMA assessments completed; all available data used from participants attending any session	Any between-session support was linked to greater increases in mindful attention and small mental health benefits versus no support; self-compassion increased in all conditions; support slightly improved attendance without reducing acceptability

Authors, year	Setting	Population (N, condition, age)	Study design	Target outcomes	Theoretical or tailoring framework	Missing data or data completeness	Key outcome pattern
Reininghaus et al. 2024 and Postma et al. 2024	Netherlands; secondary mental health services and general population	N=174 youth aged 12 to 26 with low self-esteem and childhood adversity; mean age 20.7	Assessor-blinded multicenter 2-arm parallel-group randomized clinical trial; postintervention and 6-month follow-up	Distal outcomes: improved global self-esteem, reduced psychopathology, and improved quality of life; proximal outcomes: improved momentary self-esteem and affect	Transdiagnostic ecological momentary intervention based on CBT principles targeting self-esteem	87.9% provided primary outcome data postintervention, 80.5% at 6 months, and 91.4% at either post or follow-up; mixed models used restricted maximum likelihood under MAR	SELFIE improved global self-esteem and showed beneficial signals for positive/negative self-esteem, schematic self-beliefs, momentary self-esteem/affect, general psychopathology, and quality of life, but not observer-rated symptoms or functioning
Orzikulova et al. 2024	Large university community, daily-life field deployment	N=71 participants with high smartphone overuse risk and high-quality data; age 18 to 27, mean 21.8	8-week field experiment with micro-randomized trials comparing four intervention types	Distal outcome: reduced smartphone overuse; proximal outcomes: improved intervention timing/accuracy, higher receptivity, and reduced app visit	Adaptive human-in-the-loop and explainable AI	176 eligible, 127 onboarded, 49 discontinued, and 7 excluded for insufficient data coverage; analyses used 71 high-quality participants	Adaptive models outperformed static/control methods on intervention accuracy and receptivity, expliainable AI further improved both, and app visit

**Authors, year**	**Setting**	**Population (N, condition, age)**	**Study design**	**Target outcomes**	**Theoretical or tailoring framework**	**Missing data or data completeness**	**Key outcome pattern**
				frequency/usage duration			frequency decreased by about 7.0% to 8.9%
Engelskirchen et al. 2025	Germany; daily-life smartphone EMA/EMI with baseline and post assessments	N=66 young people aged 14 to 21; spectrum from minimal symptoms to self-reported anxiety/depression; 86.4% female	Randomized controlled study; pre-post EMA before and after a 6-day EMI/control training	Distal outcomes: internalizing symptoms and ER difficulties; proximal outcomes: acceptance, problem solving, dysfunctional ER, and negative affect in daily life	Emotion regulation framework emphasizing strategy-situation fit by perceived controllability	104 started, 95 completed, final analyzed N=66; EMA completion 78.4%; intervention compliance 89.1% for content days and 61.7% for training days; ITT for included participants	No clear efficacy versus control; exploratory within-intervention analyses suggested short-term affect improvement during completed training

Table 2: Study design, theory and evidence from non-controlled pilot studies

Authors, year	Setting	Population (N, condition, age)	Study design	Target outcomes	Theoretical or tailoring framework	Missing data or data completeness	Key outcome pattern
Leonard et al. 2018	United States; transitional living program shelters for homeless adolescent mothers	N=49 homeless adolescent mothers; mean age 18.54	Feasibility and acceptability study nested within the intervention arm of a pilot randomized trial	Distal outcomes: improved emotion regulation and stress management; proximal outcomes: emotion identification, mindful attention, coping, and adaptive behavioral responses in stressful situations	Extended process model of emotion regulation, CBT, and mindfulness	Readable app data for 40/49; nightly reports completed 40.0%; 97% made at least 1 self-report; sensor-triggered use limited by technical problems	High acceptability and perceived usefulness; participants reported improved emotion awareness, coping, and stress management, but sensor-triggered engagement was inconsistent
Pramana et al. 2018 and Silk et al. 2020	United States; university clinic/metropolitan recruitment	N=34 youth aged 9 to 14 with generalized, separation, and/or social anxiety disorder; mean age 11.40	Single-arm open trial of brief CBT plus adjunctive mobile intervention	Distal outcomes: reduced anxiety symptoms/disorders; proximal outcomes: CBT skill practice and anxiety coping in daily life	Cognitive behavioral therapy for child anxiety (Coping Cat/FEAR plan) with gamification	34 enrolled, 30 completed treatment, 29 completed 2-month follow-up; app used about 12 times between sessions; no formal imputation reported	High feasibility, usability, and acceptability; youth engaged frequently with the app, especially interactive modules; brief CBT and SmartCAT was associated with improved anxiety outcomes and CBT skills, and the

Authors, year	Setting	Population (N, condition, age)	Study design	Target outcomes	Theoretical or tailoring framework	Missing data or data completeness	Key outcome pattern
							gamified version was used more than the earlier nongamified version
Neal-Barnett et al. 2019	United States; 2 urban low-income middle schools in a large Midwestern school district	N=72 Black or biracial girls in grades 7 to 8; age 12 to 15	Mixed-methods open trial; pre-post plus day 1 to day 7 in-app data	Distal outcome: reduced anxiety; proximal outcome: reduced negative thinking via musical cognitive restructuring	Musical cognitive restructuring	Sample size varied across analyses because app-server connectivity problems prevented matching some app data; no imputation reported	Negative thinking was lower at day 7 than day 1, anxiety decreased pre to post, and focus groups described calmness, behavior change, help in stressful home situations, and more focused thinking
Shrier et al. 2020	United States; 2 adolescent/young adult clinics affiliated with an urban children's hospital in the Northeast	Female clinic patients aged 15 to 24 with PHQ-9 ≥5, weekly penile-vaginal sex, and recent	Single-arm pilot of counseling plus 4-week EMI with 3-month follow-up	Distal outcomes: reduced pregnancy/STI risk behavior; proximal outcomes: reduced depressive symptoms, improved motivation/confidence to change risk behavior, and	Behavior-Determinants-Intervention logic model with motivational interviewing and cognitive-behavioral skills	N=15 completed EMI, 14 booster, and 14 completed 3-month follow-up; app engagement remained high across 4 weeks; no	Depressive symptoms and confidence improved post-EMI, depressive symptoms remained lower at 3 months, sex

Authors, year	Setting	Population (N, condition, age)	Study design	Target outcomes	Theoretical or tailoring framework	Missing data or data completeness	Key outcome pattern
		pregnancy/STI risk; N=15		improved cognitive restructuring self-efficacy		formal imputation reported	frequency decreased, and condom-unprotected sex declined by follow-up
Rauschenberg et al. 2021	Netherlands; secondary mental health services	N=11 help-seeking youth aged 14 to 25 with psychotic, depressive, or anxiety symptoms; mean age 20.3	Uncontrolled phase 1 pilot; baseline, postintervention at 3 weeks, and 4-week follow-up	Distal outcomes: psychotic, depressive, and anxiety symptoms and general psychopathology; proximal outcomes: stress sensitivity, negative affect, psychotic experiences, threat anticipation, and positive affect	Transdiagnostic compassion-focused intervention	N=10 completed postintervention and follow-up; 467/1260 EMA signals completed (37.1%)	Feasible and safe, with preliminary improvements in stress sensitivity, momentary affect, psychotic experiences, threat anticipation, and symptom outcomes
Wu et al. 2024	Country and setting not listed	N=25 participants with problematic smartphone use; mean age=22	Within-subject field experiment comparing MindShift, MindShift-Simple, and baseline over 5 weeks	Distal outcome: reduced problematic smartphone use; proximal outcomes: higher intervention acceptance, reduced app opening/usage, and improved self-efficacy	Dual systems theory and Existence, Relatedness, and Growth theory	N=31 recruited, 25 completed, 6 dropped out	MindShift showed the strongest effects on intervention acceptance and reductions in problematic smartphone use, with lower

Authors, year	Setting	Population (N, condition, age)	Study design	Target outcomes	Theoretical or tailoring framework	Missing data or data completeness	Key outcome pattern
							smartphone addiction scale scores and higher self-efficacy than baseline
van Asselt et al. 2025	Netherlands; outpatient departments of 2 mental health care facilities	N=24 autistic adolescents aged 12 to 18; mean age 15.0	Single-case experimental design (ABA) pilot with control, pre-test, post-test, and 4-week follow-up	Distal outcomes: reduced perceived stress and improved quality of life; proximal outcomes: more adaptive and less maladaptive coping	Stress recognition and adaptive coping support	N=24 at control, 23 pre-test, 17 post-test, 16 follow-up; adherence not checked; completers-only analyses used	No significant effects on stress, coping, or quality of life at post-test or follow-up

Table 3: Study design, theory, and evidence from qualitative studies

Authors, year	Setting	Population (N, condition, age)	Study design	Target outcomes	Theoretical or tailoring framework	Missing data or data completeness	Key study observations
Shrier et al. 2017	United States; 3 primary care clinics affiliated with an urban pediatric hospital	N=16 depressed young women with sexual risk behavior; mean age 19.6	Qualitative formative interview study of a proposed counseling plus EMI intervention	Distal outcomes: reduced sexual risk behavior; proximal outcomes: improved affect regulation/recognition, safer-sex self-efficacy, and healthier responses to negative mood	Cognitive behavioral therapy	NA	Participants viewed the approach as acceptable, therapeutic, and supportive; they wanted highly personalized, positive messages with flexible options and minimal repetition
Lucas-Thompson et al. 2020 (same intervention as Lucas-Thompson et al. 2023)	United States	N=22 adolescents aged 12 to 18 in Learning to BREATHE group program	Mixed-method development/content paper	Distal outcomes: reduced stress, anxiety, and internalizing symptoms; proximal outcomes: increased mindfulness, home practice, skill transfer, and use of mindfulness during high stress	Mindfulness, Information–Motivation–Behavioral Skills model/self-efficacy-informed message design	NA	Development paper describing a multi-method adaptive supplement to Learning to BREATHE, see Table 6
Ranney et al. 2021	United States; urban pediatric	N=19 adolescents aged 13 to 17 with past-year cybervictimization;	Iterative qualitative development/feasibility	Distal outcomes: reduced cybervictimization and related mental health	Motivational interviewing and cognitive	N=2 withdrew because of phone loss/breakage; 88.34% of daily	Acceptability and engagement improved across iterations;

Authors, year	Setting	Population (N, condition, age)	Study design	Target outcomes	Theoretical or tailoring framework	Missing data or data completeness	Key study observations
	primary care clinic in the Northeast	mean age 15.0; majority Hispanic and low socioeconomic status	study with 3 consecutive cohorts	consequences; proximal outcomes: improved coping, de-escalation, emotion regulation, self-efficacy, and prosocial online behavior	behavioral therapy	queries received a reply; 84% completed postintervention interviews	participants described the program as caring, helpful, motivational, and relevant, and wanted broader online-drama content, more personalization, positive tone, and quick/relevant support
Hiller et al. 2025	Germany; AI4U living laboratory	N=27 young people aged 14 to 25, generally healthy	Qualitative process evaluation embedded in 2 microrandomized trials; 16 interviews and 3 focus groups	Distal outcomes: improved mental health and emotional resilience; proximal outcomes: greater emotional self-awareness, emotion regulation, stress management, and health-promoting routines	Participatory living lab approach with AI-based personalization	27/114 completers from two trials were included; sample skewed toward highly educated participants with uneven gender distribution	Participants reported greater emotional self-awareness through EMA and better emotion regulation/stress management through EMI; valued anonymity and support, but found alarms, fixed timing, and daily-life

**Authors, year**	**Setting**	**Population (N, condition, age)**	**Study design**	**Target outcomes**	**Theoretical or tailoring framework**	**Missing data or data completeness**	**Key study observations**
							integration burdensome, and wanted more autonomy and flexibility
van Asselt and Roke 2025	Netherlands mental health outpatient services	Client panel N=15 autistic adults with IQ >85 and low to moderate support needs; practitioner focus groups N=8	Retrospective qualitative co-creation/development paper	Distal outcomes: reduced daily stress and better stress management; proximal outcomes: improved stress recognition, insight into stress patterns, and use of stress-reduction strategies	Design Thinking	NA	Co-creation produced desired intervention components (see Table 6); users wanted simplicity, predictability, and flexibility, and fixed schedules were burdensome

Table 4: Tailoring mechanisms of JITAIs evaluated in randomized controlled trials

Authors, year	Intervention options	Tailoring variables and decision rules	Decision points	Human support	Passive sensing	Ethics or youth involvement
Kennard et al. 2018	Brief inpatient ASAP intervention with 4 modules plus BRITE app for daily distress ratings, coping skills, emotion regulation, and safety-plan access	Daily distress ratings determined app response; highest distress triggered personalized safety-plan/support content	Daily postdischarge distress prompt	Yes, inpatient therapists delivered ASAP, personalized the app, and made bridging calls	No	Families involved in safety planning and app was HIPAA-compliant app
van Aubel et al. 2020	Five weekly group ACT sessions plus ACT-DL app; control received weekly film sessions plus ESM only	Prompt completion triggered either a metaphor or an exercise tied to the weekly ACT theme; disturbing emotions/thoughts branched to specific acceptance/defusion exercises; on-demand exercises available	Eight semi-random prompts/day for 3 days after each weekly session, plus morning/evening questionnaires and on-demand exercises	Yes, therapist-led sessions and staff app/device support	No	NR
Suffoletto et al. 2021	Automated text mental-health check-ins, web check-ins when emotional health was low, tailored self-care support messages, psychoeducational video links, and prompts to seek care when needed	Emotional health rating determined branching; fair/poor triggered web check-in on stressors, negative effects, and self-efficacy; low self-efficacy triggered skills support and weekly follow-up; 2 consecutive weeks of poor	Adaptive check-ins over 3 months, stepped down to monthly when doing well and increased to weekly when support was needed; one	No	No	Refined with input from a college student ambassador; privacy-protection advice included in onboarding

Authors, year	Intervention options	Tailoring variables and decision rules	Decision points	Human support	Passive sensing	Ethics or youth involvement
		mental health plus low self-efficacy triggered care-seeking prompt	repeat prompt after nonresponse			
Paetzold et al. 2022 and Reininghaus et al. 2023	Hybrid EMIcompass intervention with 4 guided compassion-focused sessions plus 6-week smartphone EMI including weekly enhancing tasks, daily consolidating tasks, optional interactive tasks, and gamified progress feedback	Basic vs elaborate track allocated after week 2 based on psychologist impression and participant experience; high stress or negative affect on EMA triggered interactive tasks; weeks repeated if fewer than 1 consolidating task completed	Weekly enhancing task, daily consolidating task at user-set time, on-demand tasks anytime, and optional EMA 6 times/day on 3 consecutive days/week with interactive tasks after qualifying EMA	Yes, trained psychologists delivered sessions with weekly feedback and email/phone support	No	NR
Schenkel et al. 2022	SMS receptivity check, choose 1 of 2 preselected if-then plans, visualize the chosen plan, then later rate stress by SMS	Baseline selection of 2 of 9 predefined plans; on a self-identified stress day, receptivity reply triggered plan choice and visualization; no reply within 6 hours stopped later messages	On one self-identified stress day, 1 hour before leaving home or at 06:00; stress rating 11 hours after first message	No	No	NR
Marciniak et al. 2023	Imager app with EMA plus mental imagery training, stress-triggered reminders, and evening imagery training	Three imagery trainings/day linked to EMA prompts; self-triggered training available; stress rating ≥4/7 triggered a	10 EMA prompts/day between 8:30 a.m. and 11:00 p.m.; imagery windows at 10:00 a.m., 2:30 p.m., and 7:00	No	No	Stakeholder students informed app design; privacy concerns discussed in development

Authors, year	Intervention options	Tailoring variables and decision rules	Decision points	Human support	Passive sensing	Ethics or youth involvement
		reminder to keep thinking in positive mental images	p.m.; evening training in final prompt			
Bell et al. 2023	Mello app with 3 daily check-ins and 12 brief CBT-based microinterventions; control had no app	Algorithm recommended 1 of 12 microinterventions based on repetitive negative thinking level, mood, activity, and location; rules matched stuck thinking, mood valence, active/passive activity, and home/away context	Randomized prompts 3 times/day between 9 AM and 9 PM; activities also available on demand	Yes, weekly brief phone calls for encouragement and troubleshooting; no therapeutic support	No	User-centered design with input from young people with lived experience and clinicians
Lucas-Thompson et al. 2023 (same intervention as Lucas-Thompason et al. 2020)	Learning to BREATHE group program plus randomized between-session supports: online library, intervention messages, just-in-time stress support, or full L2B PLUS	Intervention messages were based on the previous week's lesson; just-in-time support was triggered when mindful attention was 5 or lower or stress was 6 or higher	Intervention messages 5 times/day on non-session days; just-in-time support used 2 EMA prompts/day and triggered immediate support after qualifying responses	Yes, facilitator-led group sessions	No	NR
Reininghaus et al. 2024 and Postma et al. 2024	Blended 6-week EMI with 3 face-to-face sessions, 3 email contacts, and app-based adaptive tasks including enhancing, consolidating, and interactive tasks	App content was tailored to moment, person, and context using week-specific self-esteem content and daily-life app tasks; exact interactive triggering rules are not fully	Sessions in weeks 1, 3, and 5; email contacts in weeks 2, 4, and 6; app-based tasks delivered throughout daily life during the 6-week intervention	Yes, trained mental health professionals delivered sessions and supported engagement	No	Stakeholder/participant process-evaluation input reported

Authors, year	Intervention options	Tailoring variables and decision rules	Decision points	Human support	Passive sensing	Ethics or youth involvement
		specified in the main trial paper				
Orzikulova et al. 2024	Typing-based friction intervention requiring entry of 12 digits before continuing to a monitored app, with optional explanations and feedback prompt	Passive sensing and app-use features fed personalized machine learning models; intervention triggered when overuse was predicted; user feedback updated models daily with recency weighting; SHAP feature categories generated explanations	At monitored app launch and every 5 minutes during app use; 10-minute cool-down after intervention; daily model updates overnight	No	Yes, passive sensing and app-usage tracking via AWARE and custom logging	Privacy concerns contributed to some withdrawals; ethical/privacy risks of AI-based interventions discussed
Engelskirchen et al. 2025	Smartphone EMI with 1 reminder day, 3 days psychoeducation, and 3 days practical training in acceptance/problem solving; control received matched thought-observation training	Perceived controllability guided strategy choice: acceptance for less controllable situations, problem solving for more controllable situations	5 semi-random EMA prompts/day for 3 days pre and post; practical training self-triggered during negative situations across days 5 to 7; evening daily questionnaire	No	No	NR

Table 5: Tailoring mechanisms of JITAIs evaluated in non-controlled pilot studies

Authors, year	Intervention options	Tailoring variables and decision rules	Decision points	Human support	Passive sensing	Ethics or youth involvement
Leonard et al. 2018	Calm Mom app with nightly reports, self-initiated reports, and sensor-triggered alerts, paired with a wearable sensorband	Individual EDA threshold triggered alerts; emotion valence guided affirmations vs coping/problem-type content	Nightly report at 9 PM, self-reports anytime, and real-time alerts when EDA exceeded threshold	Yes, weekly in-person group sessions plus setup/support	Yes, wearable sensorband measuring electrodermal activity	NR
Pramana et al. 2018 and Silk et al. 2020	SmartCAT app plus clinician portal as adjunct to in-person brief CBT, with skills coach, interactive games, exposure/home challenge tasks, rewards, and secure messaging	Therapists assigned weekly session-specific modules based on treatment progress; prompts were scheduled by therapist/child and could also be self-initiated; optional geofenced anxiety locations triggered skills coaching	Daily scheduled prompts, self-initiated use anytime, and additional prompts at anxiety-provoking locations when geofencing was enabled	Yes, trained CBT therapists delivered treatment, assigned modules, monitored data, sent messages, and managed rewards	Yes, optional geofencing/location awareness	User-centered design with therapist and child input during development
Neal-Barnett et al. 2019	BYOTS app plus 8-session culturally infused SUN sister circle; girls created and recorded a personal theme song	In-app negative-thinking score determined whether to listen to the theme song; after listening, the app gave praise or told the user to replay	3 push alerts/day for 1 week, plus self-initiated use during negative thinking	Yes, part of SUN sessions, where the program taught and practiced app use	No	Participants picked song of choice as input into intervention, however no other co-design of overall intervention

Authors, year	Intervention options	Tailoring variables and decision rules	Decision points	Human support	Passive sensing	Ethics or youth involvement
Shrier et al. 2020	Personalized messages encouraging healthy behavior and CBT skill use	EMA/diary reports of negative affect, contraceptive and condom self-efficacy, pregnancy desire, desire for sex, and recent sex triggered	3 quasi-random prompts/day plus 1 daily diary at a user-set time; messages appeared automatically after qualifying responses	Yes, clinic staff delivered counseling/booster and research staff trained participants	No	Iteratively refined with participant interviews and EMI pretesting; waiver of parental permission
Rauschenberg et al. 2021	Weekly enhancing tasks, daily consolidating tasks, and interactive tasks, plus 3 face-to-face sessions	Interactive tasks were triggered when EMA stress, negative affect, or threat anticipation exceeded 4 on a 7-point scale	EMA prompts 7 times/day on 6 days/week; 1 daily consolidating task; 1 enhancing task/week; interactive task immediately after qualifying EMA	Yes, 3 face-to-face sessions with a trained psychologist, plus optional email/phone contact	No	NR
Wu et al. 2024	MindShift LLM-generated persuasive messages; MindShift-Simple without mental-state tailoring; baseline intent-report reminder	App-use behavior, physical context, mental state, goals, and habits informed persuasive content; strategies were matched to boredom, stress, or inertia	Triggered when a blacklisted app was opened and the user reported habitual use; messages could recur every 2 minutes until app exit	No	Yes, app-use, screen/unlock, and location data	NR
van Asselt et al. 2025	SAM Junior self-help app with brief stress questionnaires,	In-app algorithm calculated stress level from questionnaire responses; if	Questionnaires 2 to 4 times/day at 4-hour intervals, with 2-hour	No	No	Co-created with autistic adolescents,

Authors, year	Intervention options	Tailoring variables and decision rules	Decision points	Human support	Passive sensing	Ethics or youth involvement
	optional stress-reducing tips, and daily/weekly stress overviews	users opted in, a tip was tailored by location and stress level and selected from a user-chosen tip list	response window and reminder after 60 minutes; tip offered after stress scoring			including wording and app adaptations

Table 6: Tailoring mechanisms of JITAIs developed in qualitative studies

Authors, year	Intervention options	Tailoring variables and decision rules	Decision points	Human support	Passive sensing	Ethics or youth involvement
Shrier et al. 2017	Brief self-reports and responsive coping/safer-sex messages following counseling	Reports of affect, low condom self-efficacy, and desire for sex for feeling-related reasons would trigger tailored supportive and CBT-based messages	4 random prompts/day plus 1 daily scheduled report	Yes, proposed in-person counseling before EMI	No	Waiver of parental permission; participants directly informed message content, tone, and personalization preferences
Lucas-Thompson et al. 2020 (same intervention as Lucas-Thompson et al. 2023)	Learning to BREATHE group program plus on-demand library and mindfulness messages	Content matched the weekly theme; participants could self-tailor library content by theme, length, and activity type; JIT messages were triggered when stress was >70/100 or mindfulness was <30/100	Morning and evening EMA; up to 5 intervention messages/day; JIT message after elevated stress or low mindfulness	Yes, supplement to in-person group program	No	Adolescents drafted messages and provided timing feedback
Ranney et al. 2021	Brief in-clinic intervention plus 8 weeks of daily automated two-way SMS with links and on-demand keyword support	Daily ratings of how the day was going and whether online drama occurred in the past 24 hours guided responsive messages; tailoring was iteratively revised based on participant feedback	Daily SMS queries for 8 weeks, plus on-demand keyword-triggered support	No	No	Extensive adolescent input used iteratively to refine content, tone, tailoring, and delivery
Hiller et al. 2025	AI4U app over 40 days: 10-day EMA self-monitoring,	EMA ratings of affect, activities, and context	EMA 8 times/day during intro and 6 times/day	Yes, at least one face-to-face	No	Strong participatory involvement, including

Authors, year	Intervention options	Tailoring variables and decision rules	Decision points	Human support	Passive sensing	Ethics or youth involvement
	then 30-day EMA+EMI with exercises, dashboard, and at least one coaching session	informed EMI allocation; participants were repeatedly randomized to AI-based vs random EMI assignment; exact AI rules not detailed	during training; up to 210 decision points for AI-based vs random EMI allocation	coaching session; optional counseling support		peer and co-researchers in design, focus groups, and analysis
van Asselt and Roke 2025	Stress questionnaire, psychoeducation, tailored tips, peer stories, insight pages, journal, and later in-app exercises	A 4-level stress score guided tips; if opted in, tips were tailored by location, stress level, and user-selected preferences	Questionnaires 2 to 4 times/day at 4-hour intervals, with reminder after 30 minutes; optional on-demand entries	No	No; Fitbit testing was dropped	Strong lived experience involvement via autistic panel and experts by experience through codesign
