# Supplementary material for: Toward Participatory Precision Health With Co-Designed Recommendations: Systematic Review of Just-in-Time Adaptive Interventions in Adolescents and Young Adults
Source: J Med Internet Res. 2026 May 21;28:e84422. doi: 10.2196/84422 (PMC13193708; doi:10.2196/84422)
Supplement: Multimedia Appendix 3 [file jmir-v28-e84422-s003.pdf]

Data Extraction for Just-In-Time Adaptive Interventions (JITAIs) Addressing Adolescent and Young Adult (AYA) Physical Health

Contents:

Table 1: Study design, theory, and evidence from randomized controlled trials

Table 2: Study design, theory, and evidence from non-controlled pilot studies

Table 3: Study design, theory, and evidence from qualitative studies

Table 4: Tailoring mechanisms of JITAIs evaluated in randomized controlled trials

Table 5: Tailoring mechanisms of JITAIs evaluated in non-controlled pilot studies

Table 6: Tailoring mechanisms of JITAIs evaluated in qualitative studies

NR = not reported

Table 1: Study design, theory, and evidence from randomized controlled trials

Authors, year	Setting	Population (N, condition, age)	Study design	Target outcomes	Theoretical or tailoring framework	Missing data or data completeness	Key outcome pattern
Nollen et al. 2014	United States; afterschool programs in economically disadvantaged neighborhoods	N=51 low-income racial/ethnic-minority girls aged 9 to 14	Randomized pilot trial; mobile app vs written manual control; 12 weeks	Distal outcomes: healthier diet, lower screen time, and obesity prevention; proximal outcomes: increased fruit/vegetable intake, reduced sugar-sweetened beverages, and reduced screen time	Behavioral weight control principles with goal setting, planning, self-monitoring, feedback, cues to action, and reinforcement	44/51 completed week 12 (86.2%); 7 lost to follow-up; >94% completed dietary recalls; mobile users engaged on 63% of days and responded to 42% of prompts; no formal imputation reported	Mobile group showed small-to-moderate favorable trends for higher fruit/vegetable intake and lower sugar-sweetened beverage intake; no clear effects on screen time or BMI
Fortier et al. 2016 and Hunter et al. 2020	United States; pediatric hospital oncology outpatient setting	N=48 children undergoing cancer treatment, age 8 to 18; intervention n=20, attention control n=28	Randomized controlled pilot trial; 60 days	Distal outcomes: reduced cancer-related pain and improved quality of life; proximal outcomes: reduced average daily pain and fewer moderate-to-severe pain episodes	Social learning theory with self-efficacy-based cognitive and behavioral pain coping skills	53 randomized, 48 analyzed; 5 discontinued for not completing daily diaries; 61.2% diary completion overall; maximum likelihood for growth-curve analyses, listwise deletion otherwise	Average daily pain declined in both groups with no group difference, but Pain Buddy yielded fewer moderate-to-severe pain episodes

Authors, year	Setting	Population (N, condition, age)	Study design	Target outcomes	Theoretical or tailoring framework	Missing data or data completeness	Key outcome pattern
Pyky et al. 2017	Finland, Oulu conscription call-ups	N=496 young men; mean age 17.8; intervention n=250, control n=246	Parallel-group randomized controlled trial; 6 months	Distal outcomes: life satisfaction and self-rated health; proximal outcomes: physical activity and sitting	Transtheoretical Model	345/496 completed follow-up (69.6%); 151 lost; no formal imputation reported	Life satisfaction improved in both groups with no significant between-group difference; no significant overall effect on self-rated health, though men with poor baseline health were more likely to improve
Perry et al. 2017	United States; outpatient clinics at Arkansas Children's Hospital	N=34 adolescents aged 12 to 17 with persistent asthma; median age 15.4; adaptive mobile intervention N=17, paper intervention N=17	Randomized pilot clinical trial; 6 months	Distal outcomes: improved asthma control and self-management; proximal outcomes: asthma action plan use, asthma control, and self-efficacy	NR	28/34 completed follow-up (82.4%); 6/34 lost; per-protocol analysis among completers with no imputation	No overall between-group differences in ACT or self-efficacy; adolescents with uncontrolled asthma at baseline improved in the smartphone group; app satisfaction was high
Simons et al. 2018	Belgium, Flanders workplaces	N=130 lower-educated working young	2-group cluster randomized controlled trial;	Distal outcome: increased active lifestyle/physical activity; proximal	attitude-social influence-self-efficacy model,	Valid accelerometer data were 98%, 87%,	No significant intervention effects on objective PA, self-

Authors, year	Setting	Population (N, condition, age)	Study design	Target outcomes	Theoretical or tailoring framework	Missing data or data completeness	Key outcome pattern
		adults; mean age 25.0; intervention N=60, control N=70	baseline, 9-week posttest, and 3-month follow-up	outcomes: steps, MVPA, context-specific physical activity, and psychosocial determinants	intervention mapping, and self-regulation behavior change techniques	and 77% in intervention and 93%, 86%, and 75% in control across time; mixed models used all available data under MAR assumptions	reported PA, or psychosocial variables; engagement declined over time and advice was often not seen as motivating or well tailored
Fedele et al. 2021	United States; pediatric clinics	Adolescents aged 12 to 15 with poorly controlled persistent asthma and caregivers; randomized N=33 dyads, AIM2ACT intervention N=17, control N=16	Pilot randomized controlled trial with postintervention and 4-month follow-up	Distal outcomes: improved family asthma management and asthma control; proximal outcomes: asthma self-management, caregiver support, family communication, self-efficacy, and quality of life	Theory-informed dyadic asthma self-management intervention targeting helpful caregiver support in early adolescence	33 randomized; 97% retained overall and 100% retained in AIM2ACT	Asthma control improved significantly in intervention vs control, while other outcomes showed small to medium favorable effects without clear significance
King-Dowling	United States; pediatric cancer center and	N=224 survivors of childhood cancer aged 15	Randomized controlled trial of SCP-only vs	Distal outcomes: improved survivorship self-management and health-	NR	89% completed 4-month follow-up and 83% completed	Greater message engagement was associated with

**Authors, year**	**Setting**	**Population (N, condition, age)**	**Study design**	**Target outcomes**	**Theoretical or tailoring framework**	**Missing data or data completeness**	**Key outcome pattern**
et al. 2021	partnering adult academic cancer center	to 29; app N=110, survivorship careplan (SCP) only N=114	SCP+app; 16-week intervention; follow-up at 4 and 8 months	related knowledge/behaviors; proximal outcomes: SCP uptake, health awareness, motivation, and app engagement		8-month follow-up; 12% never read SCP and 8% never used the app	higher app acceptability

Table 2: Study design, theory, and evidence from non-controlled pilot studies

Authors, year	Setting	Population (N, condition, age)	Study design	Target outcomes	Theoretical or tailoring framework	Missing data or data completeness	Key outcome pattern
Spruijt-Metz et al. 2015 (KNOWME Networks pilot)	United States	N=10 overweight Hispanic adolescents; mean age 16.3; 50% female	Experimental pilot; baseline weekend vs intervention weekend	Distal outcome: reduced sedentary behavior and increased physical activity; proximal outcomes: fewer sedentary minutes and higher activity counts after prompts	Motivational interviewing	NR	Sedentary time decreased by 170.8 minutes versus baseline, and activity counts were higher after researcher SMS
Ding et al. 2016	United States	N=16 college students aged 18 to 25 in contemplation or preparation stage for physical activity change	4-week randomized pilot study; context-aware reminders vs randomly timed reminders	Distal outcome: increased walking and physical activity; proximal outcomes: greater awareness of walking opportunities and higher reminder acceptability	Fogg Behavior Model, Goal Setting Theory, and habit formation	16/19 completed; 2 withdrew for phone incompatibility and 1 for battery burden	Usability was high (SUS 80.0); context-aware reminders were more acceptable and significantly more effective for promoting other physical activities; walking effectiveness was higher but not significant
Caon et al. 2022 (same intervention	United Kingdom, Italy, and Spain;	Adolescents aged 13 to 16; eDiary analysis N=357	Descriptive intervention-use analysis within a nonrandomized	Distal outcomes: healthier eating and lower obesity risk. Proximal outcomes: fruit/vegetable intake,	Behavior Change Wheel, self-determination theory, positive	365 intervention participants, 357 analyzed; 8 excluded and 6 never used the	Higher eDiary engagement was associated with more fruit/vegetable intake and less breakfast

Authors, year	Setting	Population (N, condition, age)	Study design	Target outcomes	Theoretical or tailoring framework	Missing data or data completeness	Key outcome pattern
as Martin et al. 2020)	secondary schools		school/class-level evaluation	breakfast consumption, sugary drink/snack/fast-food habits, and eDiary engagement	psychology, and nudging	app; engagement declined over time	skipping, but app use declined over time
Cushing et al. 2021 and Cushing et al. 2025	United States; community sample	Adolescents aged 13 to 18; N=42 participants, NUDGE intervention N=21 and control N=21; predominantly female and mostly White	Nonrandomized matched controlled proof-of-concept study with secondary refinement analysis; 20 days	Distal outcome: increased moderate-to-vigorous physical activity (MVPA) and reduced sedentary time; proximal outcomes: daily MVPA and sedentary minutes	cybernetic control theory	accelerometer completeness was 799/800 expected days in 2021; both papers note small sample and nonrandomization	NUDGE increased MVPA by about 20.84 min/day; sedentary time was lower by about 82 min/day but not significant. Moderator analyses showed stronger MVPA effects with higher positive affect and energy, lower negative affect and fatigue, and higher perceived weather barriers; only energy moderated sedentary effects
Domin et al. 2022	Luxembourg, schools	N=18 healthy adolescents with low to moderate physical activity	4-week within-subject pilot (1 baseline week, 3	Distal outcome: increased physical activity and reduced sedentary behavior;	Control theory/self-regulation, informed by the	Days with >5 min missing were excluded from	Sedentary time fell significantly in week 1, then the effect was no longer detected;

**Authors, year**	**Setting**	**Population (N, condition, age)**	**Study design**	**Target outcomes**	**Theoretical or tailoring framework**	**Missing data or data completeness**	**Key outcome pattern**
		(PAQ-A ≤3); age 16 to 18; mean age 16.33	intervention weeks)	proximal outcomes: sedentary minutes, MVPA minutes, and step count	Behaviour Change Wheel	change-point analyses	personalized prompts increased heart rate in week 2 and showed marginal step effects, but overall moderate-to-vigorous physical activity and total steps did not clearly increase

Table 3: Study design, theory, and evidence from qualitative studies

Authors, year	Setting	Population (N, condition, age)	Study design	Target outcomes	Theoretical or tailoring framework	Missing data or data completeness	Key study observations
Chand et al. 2006	United States; university dining services	Design target: female college students aged 18 to 24; N=9	Qualitative service design/formative study using journals, interviews, contextual inquiry, competitive analysis, and prototype testing	Distal outcomes: healthier food choices and eating habits; proximal outcomes: improved meal selection, nutrition awareness, and use of just-in-time meal advice	Service-design and context-aware recommender concept informed by USDA dietary guidance/food pyramid	NA	Produced the Balance Pass concept integrated with campus ID meal plans (see Table 6); key limitations were assuming purchased food was eaten and incomplete capture of non-ID purchases
Fedele et al. 2020	United States; clinics in Florida and Kansas plus a national online panel	Adolescents aged 13 to 17 with persistent asthma and caregivers; interviews N=20 dyads, crowdsourcing N=257 adolescents, advisory board N=4 dyads	Iterative user-centered intervention development study	Distal outcome: improved inhaled corticosteroid adherence and asthma self-management; proximal outcomes: better self-regulation, problem solving, self-efficacy, and responsiveness to adherence barriers	Self-regulation theory and problem-solving therapy with gain-framed messaging	NA	Adolescents supported adaptive, personalized mobile support; 93% of messages were acceptable, and feedback refined tone, wording, timing, and problem-solving flow

Authors, year	Setting	Population (N, condition, age)	Study design	Target outcomes	Theoretical or tailoring framework	Missing data or data completeness	Key study observations
Martin et al. 2020 (same intervention as Caon et al. 2022)	United Kingdom, Italy, and Spain; secondary schools	Adolescents aged 13 to 16; co-design n=74	Iterative co-design and usability study	Distal outcomes: healthier eating and lower obesity risk. Proximal outcomes: healthier food choices, engagement with self-monitoring, and acceptability/usability of the eDiary	Behavior Change Wheel, self-determination theory, positive psychology, and nudging	NA	Usability improved across iterations; adolescents wanted simple logging, clear feedback, and appealing, personalized support

Table 4: Tailoring mechanisms of JITAIs evaluated in randomized controlled trials

Authors, year	Intervention options	Tailoring variables and decision rules	Decision points	Human support	Passive sensing	Ethics or youth involvement
Nollen et al. 2014	MyPal handheld app with 3 sequential 4-week modules for fruit/vegetables, sugar-sweetened beverages, and screen time, with goals, plans, prompts, tips, feedback, and song rewards	Girls set 2 daily goals and a plan for each module and self-monitored progress; responding to at least 80% of prompts earned 1 song/day	5 preselected times/day for self-monitoring; module changes at weeks 1, 5, and 9	No	No	NR
Fortier et al. 2016 and Hunter et al. 2020	Pain Buddy app with twice-daily symptom diaries, remote symptom monitoring, cognitive/behavioral pain coping skills training, and avatar-based gamified rewards	Diary symptom ratings triggered skills training and nurse practitioner alerts; alerts occurred for pain >7.5 or prespecified combinations of frequency, severity, and distress	Twice-daily symptom diaries, morning and evening; alerts triggered immediately when thresholds were met	Yes, nurse practitioner received symptom alerts and research staff supported training/adherence	No	Adolescents involved during intervention development including avatar selection
Pyky et al. 2017	MOPOrtal mobile service with physical activity goals/guidance, tailored feedback, weekly tailored messages, Clans of Oulu game, and social features	Stage of exercise behavior change rated at baseline and monthly; weekly messages tailored to stage	Baseline and monthly stage rating; weekly messages; activity uploads at least every 3 weeks with reminder texts	No	Yes: Polar Active monitor tracked activity and sitting	Young men were involved in iterative design, development, and testing before the trial
Perry et al. 2016	Personalized smartphone asthma action plan	Entered peak flow or symptoms	Daily use plus as-needed symptom entries;	Yes, asthma education/training for	No	App refined using input from

Authors, year	Intervention options	Tailoring variables and decision rules	Decision points	Human support	Passive sensing	Ethics or youth involvement
		automatically triggered Green, Yellow, or Red zone instructions; severe symptoms triggered immediate emergency guidance	immediate feedback after each entry; daily medication reminders and twice-weekly education messages	all; research staff and caregivers received safety alerts		adolescents with asthma and caregivers
Simons et al. 2018	Active Coach app with tailored goals, tips, facts, and feedback plus Fitbit Charge	Registration data, focus choice, monitoring week activity, goal achievement, and reported barriers informed app content	End of monitoring week for goal setting; daily goal feedback; weekly goal review/adjustment; Monday and Friday tips, Wednesday facts	No	Yes: Fitbit steps and smartphone sensor tracking of active transport	Developed with consultation and pretesting with the target group
Fedele et al. 2021	Feedback on asthma management strengths/weaknesses, goal setting, behavioral contracting, and skills-training videos	EMA identified strengths and weaknesses in asthma management; dyads then selected goals and created behavioral contracts, with updated feedback after a second assessment period	EMA twice daily during 1-week needs-assessment periods; repeated goal-setting and review cycles over 2 months	Dyadic intervention with caregiver involvement and research staff app installation/support	NR	NR
King-Dowling et al. 2021	1 to 2 tailored messages/day, links/videos, interactive content, points, and optional self-management applets	App messages tailored to age, treatment, and chosen health goal; interactive replies	1 to 2 messages daily for 16 weeks; weekly goal/adherence surveys;	Yes, staff created/reviewed survivorship care plans	No, but optional syncing with	NR

Authors, year	Intervention options	Tailoring variables and decision rules	Decision points	Human support	Passive sensing	Ethics or youth involvement
		triggered automated follow-up content	outreach after 2 weeks inactivity	and followed up with inactive users	Fitbit/Apple Health	

Table 5: Tailoring mechanisms of JITAIs evaluated in non-controlled pilot studies

Authors, year	Intervention options	Tailoring variables and decision rules	Decision points	Human support	Passive sensing	Ethics or youth involvement
Spruijt-Metz et al. 2015 (KNOWME Networks pilot)	Wearable heart-rate/activity sensors, smartphone sedentary-analyzer app, and server dashboard; automated MOVE alert plus SMS dialogue	If 2 hours of continuous sedentary behavior occurred, the app sent a MOVE alert; if no response within 10 minutes, researchers sent motivational texts tailored using intake/context information	Continuous sensing with trigger at 2 hours sedentary and SMS follow-up after 10 minutes of nonresponse	Yes, researchers monitored the dashboard and sent SMS	Yes, wearable heart-rate/activity sensors; ActiGraph used for measurement	User-centered design with youth input
Ding et al. 2016	motivational walking reminders with short-term step goals	Phone use, sedentary time, walking state, and post-meal context triggered reminders	Event-triggered throughout daily life when walking opportunities were detected	No	Yes, smartphone and smartwatch sensing	NR
Caon et al. 2022 (same intervention as Martin et al. 2020)	eDiary food logging, immediate feedback, tailored suggestions, gamification, and companion app/portal	Food entries and dietary target behaviors drove immediate feedback, meal suggestions, badges/challenges, and tailored recommendations	Meal entries during daily use; once-daily reminders; evaluations at baseline, 2, 4, and 6 months	No	No	Adolescents were involved in iterative co-design and usability testing
Cushing et al. 2021 and Cushing et al. 2025	NUDGE automated text-message chatbot over 20 days: daily goal setting, next-morning reminder, end-of-day moderate-to-vigorous physical	Implemented tailoring variable was prior-day self-reported MVPA; new goals were constrained to 75% to 125% of prior MVPA, with	Participant-selected evening goal-setting/self-monitoring time, next-morning reminder, and daily	No	Yes, passive ActiGraph measurement	NR

Authors, year	Intervention options	Tailoring variables and decision rules	Decision points	Human support	Passive sensing	Ethics or youth involvement
	activity (MVPA) self-monitoring, feedback, and goal revision; control completed comparable procedures without intervention content	modified feedback when users exceeded 60 min/day	feedback/goal revision; 4 EMA prompts/day were measurement only			
Domin et al. 2022	Fitbit app plus Charge 4 tracker with daily personalized physical activity prompts, move reminders, graphs/stats, rewards, and chat-based assistance	Prompts were tailored to daily step progress relative to goal using 5 achievement bands; weekly goals were adjusted by 5% based on prior-week performance	Daily prompt after school (5 PM to 7 PM); move reminders hourly if sedentary after school (4 PM to 9 PM); weekly goal review	Yes, researcher remotely set goals, monitored data, and provided support	Yes, Fitbit passive sensing	Prompt templates informed by adolescent focus-group input

Table 6: Tailoring mechanisms of JITAIs developed in qualitative studies

Authors, year	Intervention options	Tailoring variables and decision rules	Decision points	Human support	Passive sensing	Ethics or youth involvement
Chand et al. 2006	ID card meal tracking with meal advice, dietary history, and reward points for healthier choices	Purchase history approximated eating habits; campus location informed nearby options; recommendations compared meals by nutrition, price, and time, and food-history feedback was pushed at purchase	At ID card swipe/purchase, on demand when students requested meal advice, and periodically through reward accumulation	No	Yes, ID card purchase tracking and campus location context	Target users contributed through participatory journals, interviews, contextual inquiry, and think-aloud testing
Fedele et al. 2020	Tailored text messages, videos, goal-setting content, and problem-solving modules	Inhaled corticosteroids adherence <80% triggered intervention; motivation determined goal setting vs education, and reported barriers determined problem-solving content	Triggered when adherence dropped below threshold, with follow-up based on motivation and barriers	No	Yes, Bluetooth-enabled inhaler sensor for medication monitoring	Extensive adolescent and caregiver involvement through interviews, crowdsourcing, and advisory boards
Martin et al. 2020 (same intervention as Caon et al. 2022)	eDiary food logging, immediate feedback, tailored suggestions, gamification, and companion app/portal	Food entries and dietary targets drove immediate feedback and tailored suggestions; design was iteratively refined with adolescents	Meal entries during daily use; once-daily reminders in the intervention design	No	No	Adolescents were involved in iterative co-design and usability testing
